# A case report of a gastric perforation in a giant inguinoscrotal hernia: A two-step approach

**DOI:** 10.1016/j.ijscr.2019.01.041

**Published:** 2019-02-01

**Authors:** Paul Sayad, Audrey Zhen Tan

**Affiliations:** Department of General Surgery, HealthBay Polyclinic, Dubai, United Arab Emirates

**Keywords:** Hernia, Giant hernia, Inguinoscrotal hernia, Gastric perforation, Case report

## Abstract

**Introduction:**

An inguinoscrotal hernia is defined as giant if descending below the midpoint of the inner thigh of a patient in upright position. It is an uncommon condition and rarely encountered in clinical practice that can lead to severe complications such as gastric perforation.

**Presentation of case:**

We present a case of a 50-year old male suffering from a gastric perforation in a giant inguinoscrotal hernia that was managed in a two-step approach.

**Discussion:**

In our patient, we opted for a two-step approach. The initial phase consisted in the release of the stomach, repair of the perforation, the control of the infection and the stabilization of the patient. The repair of the hernia was performed uneventfully three months later in the second phase.

**Conclusion:**

In a giant inguinal hernia with gastric perforation, delaying the hernia repair when possible can decrease the complexity of the procedure and most likely its morbidity and mortality.

## Highlights

• Gastric perforation in a giant inguinal hernia is a rare and morbid complication.
• Repair of a giant inguinal hernia complicated by gastric perforation should be delayed when possible.
• Gastric rupture in a giant inguinal hernia is the result of the downward pulling exerted by the hernia content.

## 1 Introduction

An inguinoscrotal hernia is defined as giant if descending below the midpoint of the inner thigh of a patient in upright position [1]. It is an uncommon condition and rarely encountered in clinical practice. Severe complications such as duodenal [[2], [3], [4], [5]] and gastric [[6], [7], [8]] perforation have been reported with high morbidity and mortality rate. In line with the SCARE criteria [9], we are presenting a case report of a gastric perforation in a giant inguinoscrotal hernia which was managed surgically in a two-step approach.

## 2 Presentation of case

**Fig. 1:**
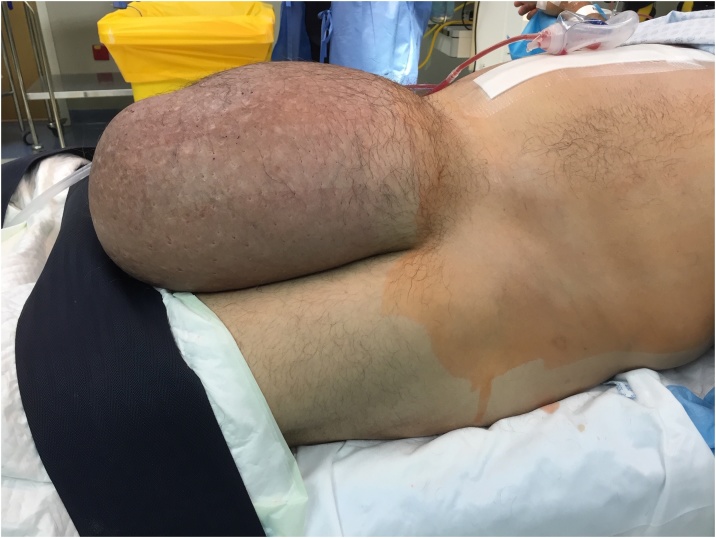
Giant inguinoscrotal hernia.

**Fig. 2:**
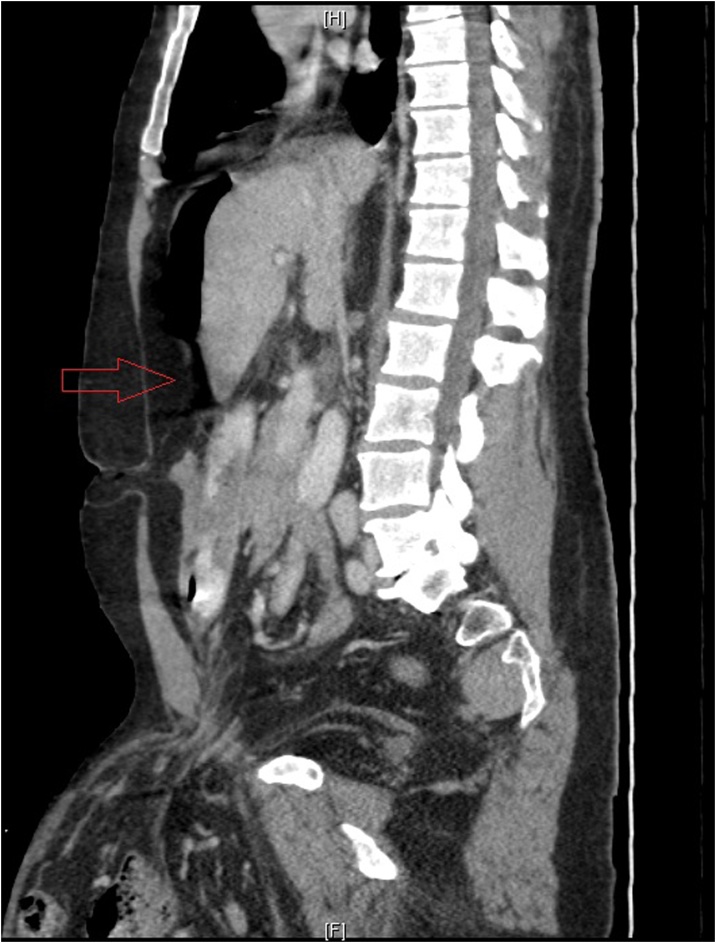
CT scan of the abdomen. free air (arrow).

A 50-year old male, visiting Dubai from his home country in South Africa was admitted through the emergency room with sudden severe abdominal pain and swelling. He had as well a large amount of hematochezia. The patient had a history of chronic giant inguinoscrotal hernia (Fig. 1) for over 10 years reaching just above the knee level. A computed tomography (CT) scan of the abdomen revealed the presence of free air and free fluid in the abdomen (Fig. 2). The patient was taken to the operating room the same day and a mid-line laparotomy was performed revealing the presence of a large amount of gastric content and pus in the abdominal cavity. A 4 cm tear in the anterior wall of the stomach was identified.

**Fig. 3:**
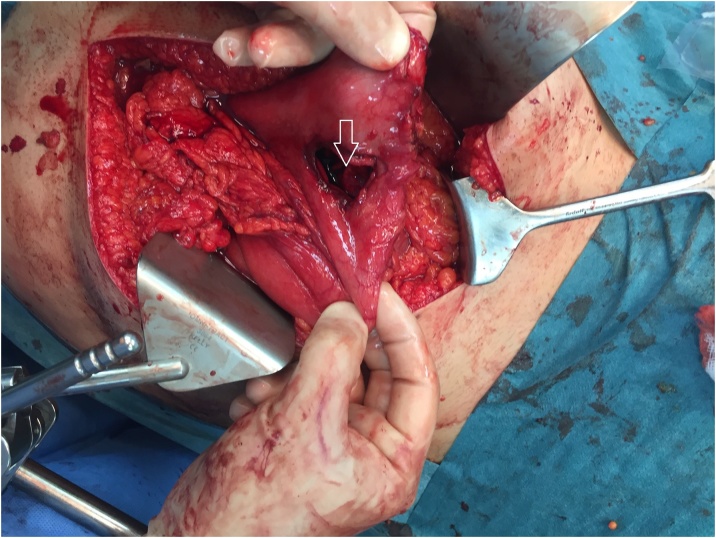
Gastric perforation. (arrow).

**Fig. 4:**
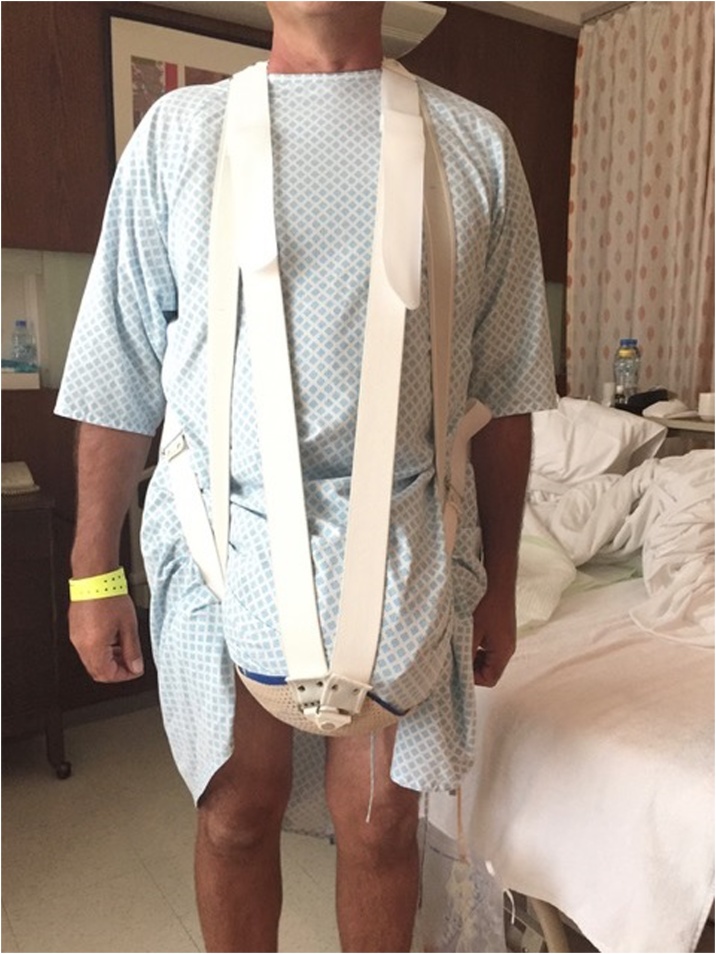
Customized inguinoscrotal support.

The stomach was being pulled down by the large hernia, which had caused the tear. The stomach was released by transecting the omentum at the level of the greater curvature allowing a repair of the perforation without any tension (Fig. 3). The 4 cm tear was repaired using 2-0 Vicryl running suture. The abdomen was irrigated with several liters of normal saline solution and suctioned until the irrigation fluid was clear. A drain was left in the subhepatic area. The fascia was closed using #1 PDS running suture and the skin was closed using staples. The patient was transferred back to the intensive care unit. He was kept NPO, started on parenteral nutrition and placed on Meropenem and Fluconazole IV. After initial signs of sepsis, the patient’s condition improved with the decrease of the fever and the C-Reactive Protein (CRP). On post-operative day 6, he started having again fever and increased CRP. A CT scan of the abdomen showed a large fluid collection in the scrotal hernia sac. He underwent an ultrasound guided drainage of the collection. On post-operative day 7, the patient was transferred to the ward. The bowel function had returned to normal. His nasogastric tube was removed and he was started on oral diet. Continuous drainage was done until the infection had cleared and the CRP normalized. The patient was discharged home on post-operative day 18 with the plan to perform a delayed hernia repair. An inguinoscrotal support device was custom made (Fig.4) to prevent the pulling of the organs and further complications. Since the patient was on a visit to Dubai, he preferred to fly back to his home country for the hernia repair.

The operation was performed 3 months later through an inguinal incision and was uneventful. He recovered without complications or signs of recurrence on his 6 months follow- up.

## 3 Discussion

Giant inguinal hernia complicated by gastric perforation is quite a challenging situation. In addition to the closure of the perforation the surgeon is faced with the repair of the giant hernia which adds complexity and severe morbidity to the operation.

In our case, soon after the abdominal cavity was explored and the stomach and bowel were inspected, the mechanism of perforation became more obvious. In fact, the gastric rupture was the result of the downward traction exerted by the heavy content of the hernia on the stomach and not secondary to an incarceration. The bowel was not strangulated as well. Ishii et al. [2] describe a similar mechanism in their report of a duodenal perforation secondary to a giant inguinal hernia. This was probably as well the case in the report of gastric perforation by Fitz et al [7] where the entire bowel was found to be viable. Most morbidities and mortalities reported in the literature were related to the complications that are secondary to the acute increase in intra-abdominal pressure after placing the hernia content back in the abdomen during a concomitant hernia repair [2,3].

In our patient we opted for a two-step approach. The initial phase consisted in the repair of the perforation, the control of the infection and the stabilization of the patient. The delayed hernia repair was performed in the second phase. By doing so, we were able to avoid an initial repair of a complex hernia in an unstable patient with a severely contaminated abdomen where the intra-abdominal pressure is already increased secondary to the peritonitis and the ileus.

## 4 Conclusion

In a giant inguinal hernia with gastric perforation, delaying the hernia repair when possible can decrease the complexity of the procedure and most likely its morbidity and mortality.

## Conflicts of interest

The authors certify that we have no affiliations with or involvement in any organization or entity with any financial interest or non-financial interest in the subject matter or materials discussed in this manuscript.

## Sources of funding

None.

## Ethical approval

For this case report, our institute exempted us to take ethical approval as this is not a research study.

## Consent

Written informed consent was obtained from the patient for publication of this case report and accompanying images. A copy of the written consent is available for review by the Editor-in-Chief of this journal on request.

## Author contribution

Paul Sayad performed the exploratory laparotomy to control the bleeding and repair of gastric perforation, revised the paper and made the literature review. Audrey Zhen Tan drafted the manuscript and consolidated the source materials for this case report.

## Registration of research studies

Not applicable.

## Guarantor

Dr. Paul Sayad.

The manuscript has been read and approved by all the authors and is not under consideration for publication elsewhere.

## Provenance and peer review

Not commissioned, externally peer-reviewed.
